# Photoactivatable Alkyne Tag for Photolabeling Biomolecules in Living Cells

**DOI:** 10.1002/cbic.202500190

**Published:** 2025-05-26

**Authors:** Yuki Umeda, Hao Zhu, Satoshi Yamaguchi, Sho Nakamura, Masato Takada, Shin Izuta, Akimitsu Okamoto

**Affiliations:** ^1^ Department of Chemistry and Biotechnology Graduate School of Engineering The University of Tokyo 7‐3‐1 Hongo, Bunkyo‐ku Tokyo 113‐8656 Japan; ^2^ SANKEN Osaka University 8‐1 Mihogaoka, Ibaraki‐shi Osaka 567‐0047 Japan

**Keywords:** alkyne‐forming reaction, cholesterol analog, light‐induced molecular imaging, photoactivatable alkyne tag, photodegradable protecting group

## Abstract

Light‐induced molecular imaging methods have attracted considerable attention owing to their potential for monitoring changes in the localization of intracellular molecules, which can provide valuable insights into the molecular mechanisms of living systems. In this article, a photoactivatable alkyne tag is developed by modifying an unstable intermediate of the alkyne‐forming reaction with a photodegradable protecting group; the photodegradation triggers the conversion of the intermediate into a linear alkyne in an aqueous solution. The developed photoactivatable alkyne tag is incorporated into a cholesterol analog, introduced into living cells, and exposed to a biocompatible dose of 365 nm light. Subsequently, the cholesterol analog in light‐irradiated cells is microscopically visualized through alkyne‐specific biotinylation via copper‐catalyzed azide–alkyne cycloaddition and biotin‐specific labeling with fluorescence‐labeled streptavidin. The obtained results indicate that the photoactivatable alkyne tag can be photoconverted into alkyne derivatives inside cells and applied to the light‐induced intracellular imaging of biomolecules. This photoactivatable chemical tag can potentially expand the range of applications of light‐induced molecular imaging of various biomolecules.

## Graphical abstract

A photoactivatable linear alkyne tag is developed by caging the intermediate of the Eschenmoser–Tanabe reaction to expand photoinduced intracellular molecular imaging to a variety of biomolecules. The photoactivatable tag is modified onto a cholesterol analog and introduced into cells. After light irradiation, the tag is successfully labeled with azide‐functionalized fluorescent dye, achieving photoinduced intracellular molecular imaging.

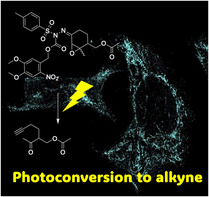

## 1 Introduction

Monitoring the dynamic localization of intracellular biomolecules can provide valuable insights into mechanisms underlying biological phenomena.^[^ ^1^, ^2^ ^]^ Thus far, diverse chemical and biological “tags” have been developed to selectively visualize biomolecules in living systems.^[^ ^3^, ^4^, ^5^, ^6^, ^7^, ^8^, ^9^, ^10^, ^11^, ^12^ ^]^ For example, tags are introduced through genetic^[^ ^13^, ^14^ ^]^ or metabolic engineering^[^ ^15^, ^16^ ^]^ into biopolymers, such as proteins and sugar chains. Further, for small biomolecules, such as nucleotides^[^ ^17^ ^]^ and lipids,^[^ ^18^ ^]^ tags are incorporated by synthesizing tagged analogs in vitro, which are then introduced into living systems. Tagged biomolecules can be visualized through modifications with fluorophores using approaches, such as immunolabeling, metal complexation,^[^ ^5^, ^6^ ^]^ protein–ligand binding,^[^ ^7^, ^8^ ^]^ bioorthogonal reactions,^[^ ^9^, ^10^, ^15^ ^]^ or enzymatic reactions.^[^ ^11^, ^12^ ^]^

Alkyne tags are attractive for bioimaging applications^[^ ^19^, ^20^, ^21^, ^22^, ^23^, ^24^ ^]^ because their small and simple chemical structure can minimize interference with the original properties of the target biomolecule. In addition, alkyne moiety can be bioorthogonally conjugated to azide‐functionalized probes via Cu(I)‐catalyzed azide–alkyne cycloadditions (CuAACs),^[^ ^20^, ^21^ ^]^ even on the surfaces of living cells and in vivo.^[^ ^22^, ^23^ ^]^ Moreover, alkyne tags enable the noninvasive visualization of molecules in real‐time through their characteristic Raman scattering spectra, thereby, eliminating the need for additional labeling or staining.^[^ ^25^, ^26^, ^27^, ^28^, ^29^ ^]^

We hypothesize that we can selectively label only tagged molecules in light‐exposed regions by making alkyne tags photoactivatable, thereby advancing the spatiotemporal studies of molecular dynamics. A photoactivatable precursor of dibenzocyclooctyne (DBCO) has been reported as a photoactivatable alkyne tag to aid in achieving the light‐guided visualization of cell surface molecules.^[^ ^30^ ^]^ DBCO reacts efficiently with azidated molecules via copper‐free cycloaddition owing to its strained cyclooctyne structure.^[^ ^31^, ^32^ ^]^ Although DBCO has been used in various applications,^[^ ^33^, ^34^, ^35^, ^36^ ^]^ its high reactivity can lead to thiol‐yne reactions under physiological conditions.^[^ ^37^, ^38^ ^]^ Thus, it is difficult to use photoactivated DBCO as a tag for selective labeling in intracellular environments with high concentrations of thiol species, such as reduced glutathione.

**Figure 1:**
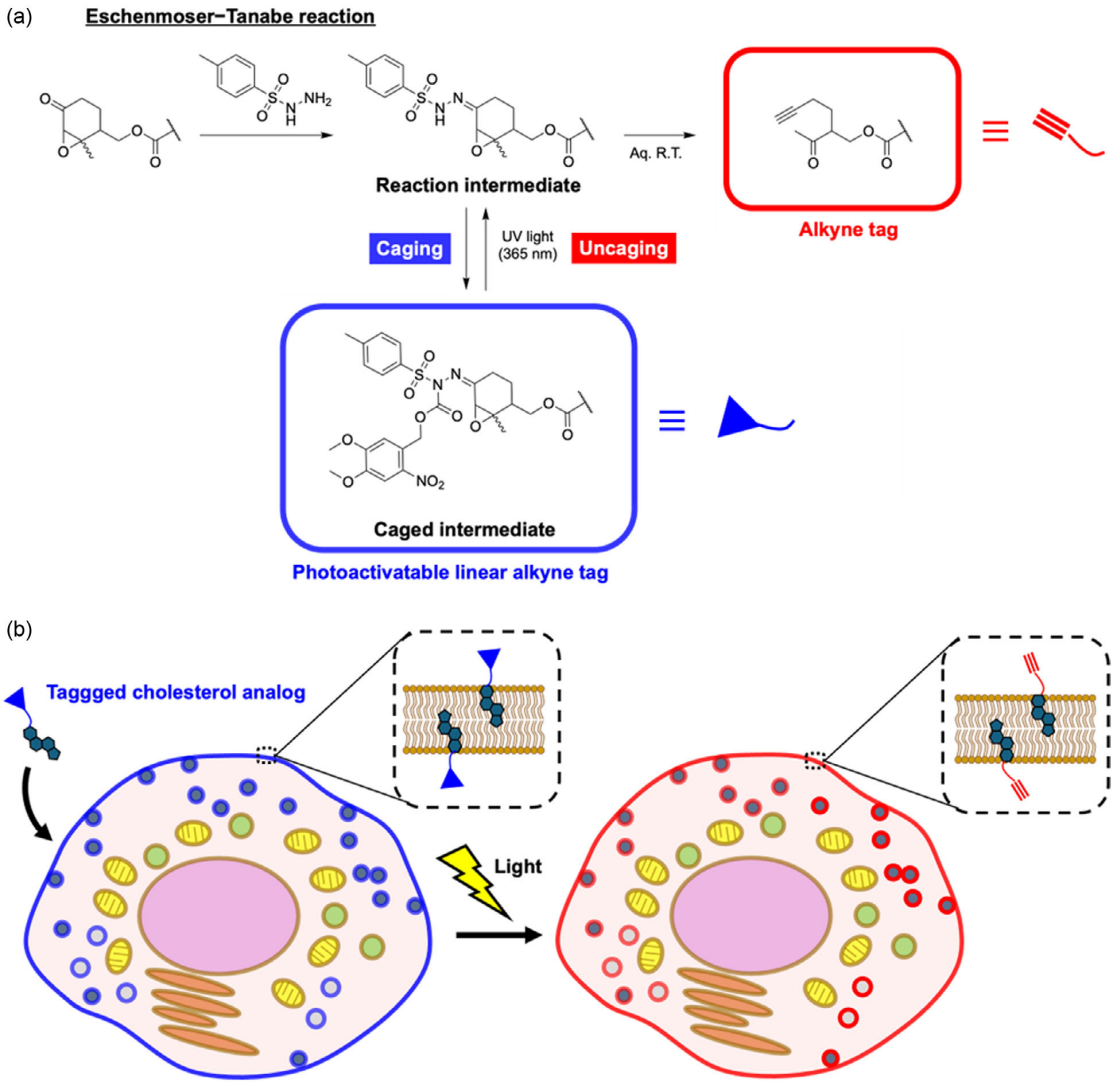
Molecular design of photoactivatable linear alkyne tag and application for intracellular photoactivation. a) Chemical structures and reaction schemes of caging of the intermediate of the Eschenmoser–Tanabe reaction and uncaging by exposure to ultraviolet light. The wavy bonds depict stereogenic centers of unknown configuration. b) Schematic of the intracellular photoactivation of tagged cholesterol analogs.

In this study, we developed a novel tag that can be photoconverted to a linear terminal alkyne under physiological conditions. Linear alkynes do not react with thiol moieties unless thiol radicals are generated through photosensitizer‐mediated reactions, which makes them beneficial for intracellular use.^[^ ^23^, ^25^, ^26^, ^27^, ^28^, ^29^, ^38^ ^]^ Further, terminal alkynes are ideal for molecular labeling because they can efficiently undergo CuAAC because of the release of the terminal proton.^[^ ^24^ ^]^ An Eschenmoser–Tanabe reaction generates linear terminal alkynes under mild conditions.^[^ ^39^, ^40^, ^41^, ^42^ ^]^ We have previously demonstrated that this reaction readily occurs in aqueous solutions (*t*_1/2_ ≤ 20 min) and can be applied to create chemically activatable alkyne tags for fluorescence^[^ ^41^ ^]^ and Raman imaging of cells.^[^ ^42^ ^]^ In these studies, we observed that the hydrazone intermediate of the Eschenmoser–Tanabe reaction was unstable in water; however, it could be stably isolated in organic solvents. Recently, molecular “caging” via modification with photolytic protective groups has become a popular strategy for converting biomolecules into photoresponsive forms.^[^ ^43^, ^44^, ^45^, ^46^ ^]^ Inspired by this approach, we aimed to cage a hydrazone intermediate with a photolytic protection group in organic solvents to synthesize a photoactivatable alkyne tag (**Figure** **1**a). This tag can then be uncaged by light and converted to alkynes in aqueous solutions. To this end, we successfully developed a photocaged alkyne precursor tag, attached it to cholesterol analog, and incorporated it into live cells. Using this system, we achieved the light‐dependent visualization of tagged cholesterol analogs within cells.

## 2 Results and Discussion

**Figure 2:**
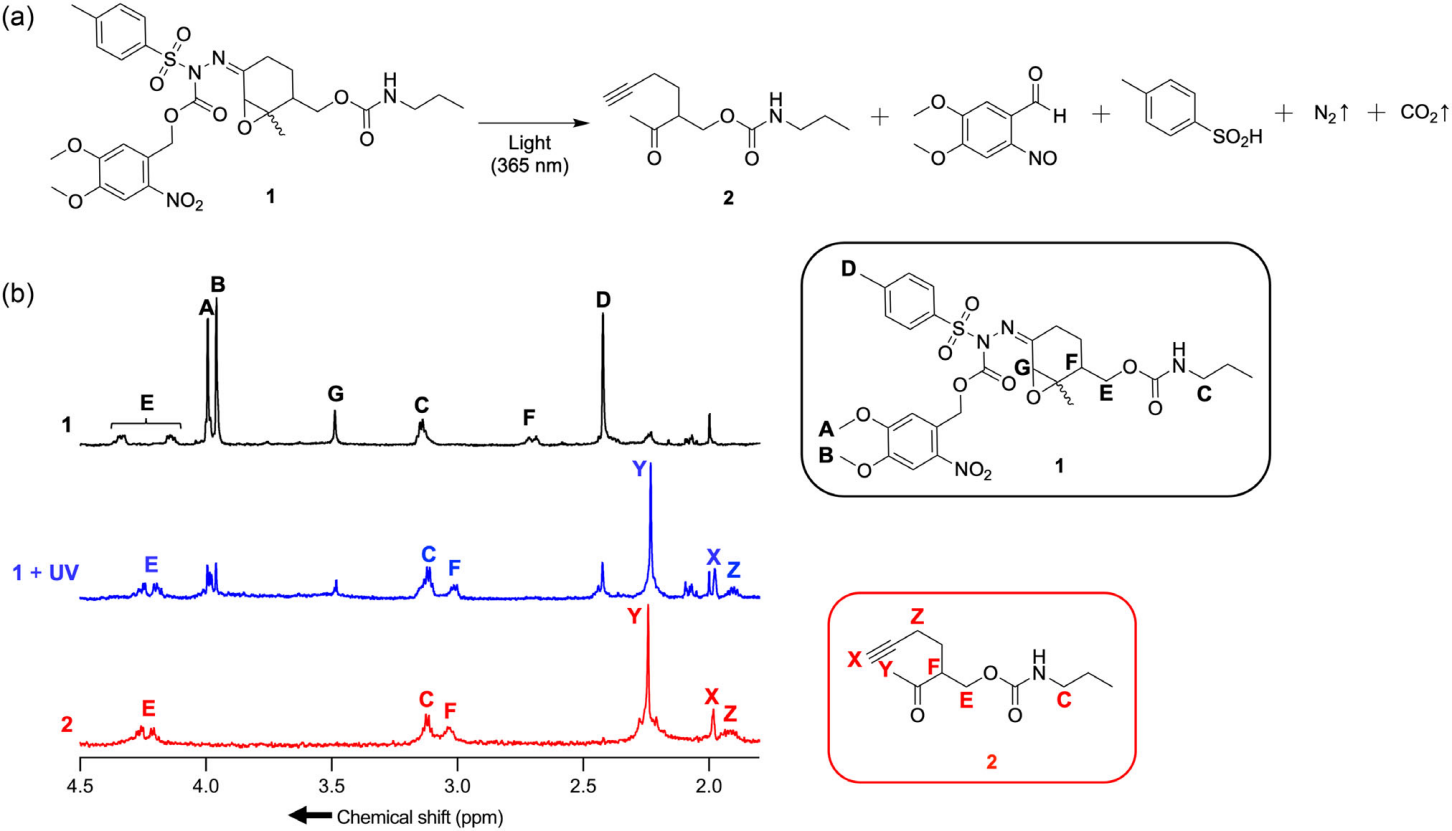
Photoconversion of the caged intermediate moiety to an alkyne moiety. a) The reaction scheme of the photoconversion of the model caged alkyneprecursor **1** to the corresponding alkyne **2**. The wavy bonds depict stereogenic centers of unknown configuration. b) ^1^H‐NMR spectra of **1** with and without light exposure. As positive control, the ^1^H‐NMR spectra of **2** was shown together. The characteristic protons on the chemical structures of **1** and **2** were named by some alphabets and represented with their positions on the chemical structures.

We first synthesized a photocaged alkyne precursor **1** as a model compound for a photoactivatable linear alkyne tag and examined its photoconversion into an alkyne compound **2** (**Figure** **2**a). Alkyne precursor **1** was prepared by reacting a starting material comprising an *α*,*β*‐epoxy cyclohexanone ring with a *p*‐toluenesulfonyl hydrazine (TsNHNH_2_) derivative (Scheme S1, Supporting Information). In this TsNHNH_2_ derivative, a photocleavable protecting group (6‐nitroveratryloxycarbonyl) was introduced to the hydrazine nitrogen atom on the side substituted with a tosyl group to yield the alkyne precursor **1** (Scheme S1, Supporting Information).

Alkyne precursor **1** was designed to undergo photodeprotection under light exposure, forming an opened heptynone derivative **2** in an aqueous solution (Figure 2a). We confirmed using proton nuclear magnetic resonance (^1^H NMR) spectroscopy that the conversion of **1** to **2** occurred after light exposure in aqueous solutions (Figure 2b). After exposure to 365 nm light in a 50% acetonitrile aqueous solution, followed by evaporation, the ^1^H NMR spectra of precursor **1** were measured in chloroform‐*d*_3_. The ^1^H NMR spectroscopic analysis revealed that, upon light exposure of compound **1**, protons E and F exhibited pronounced chemical shift changes and new signals corresponding to protons X, Y, and Z emerged, which are consistent with those of compound **2** (Figure 2b).

**Figure 3:**
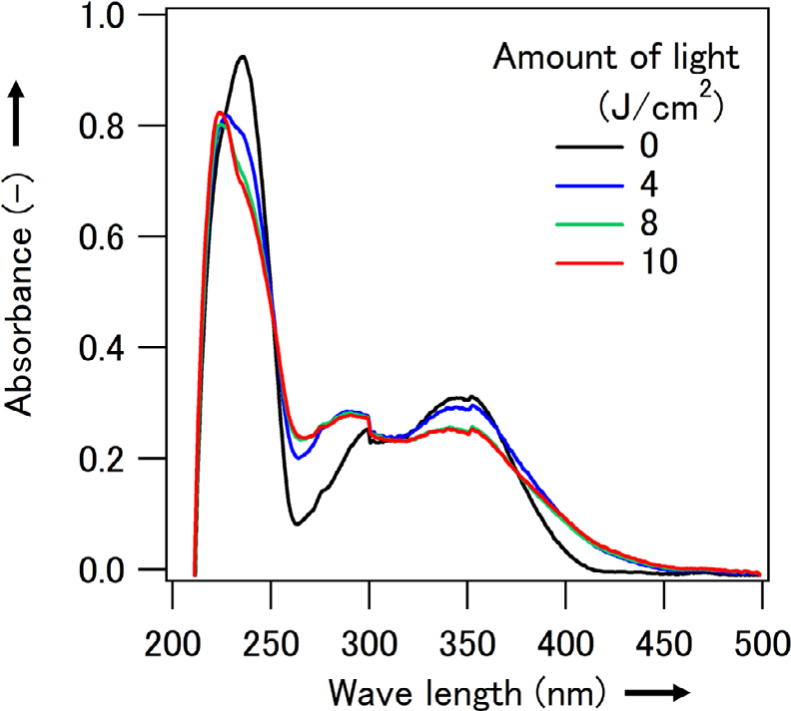
Absorbance spectra of caged alkyne precursor **1** after exposure to various amounts of light from 0–10 J cm^−2^.

The ultraviolet–visible (UV–Vis) absorption spectra of the aqueous solutions of **1** (0.1 mM) were measured after exposure to various intensities of light in the range of 0–10 J cm^−2^. Absorbance at 350 nm decreased with an increase in light intensity (**Figure** **3**). This light‐induced change saturated above 8 J cm^−2^ at the high concentration required to detect conversion from absorbance spectra. A lower concentration was required when visualizing tagged molecules in mammalian cells using a fluorescence microscope, which reduced the amount of light required. These results confirmed that alkyne precursor **1** is photoconverted to alkyne derivative **2** in an aqueous solution upon exposure to a biocompatible dose of light.

**Figure 4:**
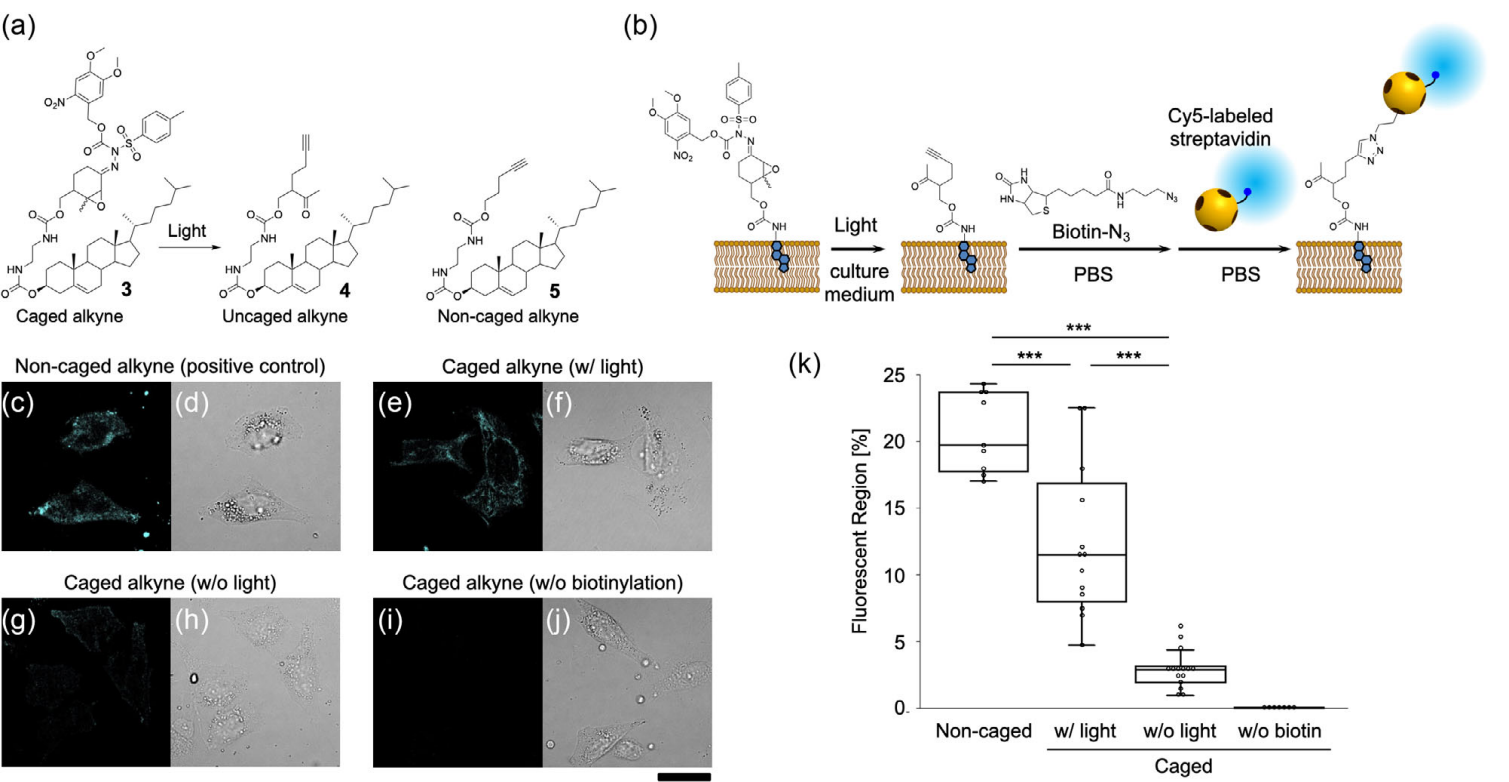
Light‐induced activation of alkyne labeling in living cells. a) Chemical structures of photocaged alkyne‐tagged cholesterol **3**, uncaged alkyne‐tagged cholesterol **4**, and noncaged alkyne‐tagged cholesterol **5**. The wavy bonds depict stereogenic centers of unknown configuration. b) Schematic of the photoactivation of **3** and fluorescence staining in cells. c–j) Confocal laser scanning microscope images of cells treated with **3** or **5** after photoactivation and fluorescence staining: c,d) Positive control cells treated with **5** after staining through biotinylation with biotin–N_3_ and subsequent binding to Cy5–SA; e,f) cells treated with **3** after photoactivation and staining with biotin–N_3_ and Cy5–SA; g,h) negative control cells treated with **3** only after staining (without photoactivation); and i,j) negative control cells treated with **3** after photoactivation and staining only Cy5–SA (without biotinylation). c,e,g,i) Red fluorescence images of Cy5 and d,f,h,j) differential interference contrast images. Scale bars: 30 μm. k) Fluorescent region per each cell in (c,e,g,i). Values and error bars represent mean ± standard deviation (*n* > 7). ***p < 0.05 (*t*‐test).

A tagged cholesterol analog **3** (**Figure** **4**a) was introduced into living cells and served as a light‐induced alkyne labeling experiment. Photocaged alkyne‐tagged cholesterol **3** was synthesized by modifying the synthesis scheme of model alkyne precursor **1** (Scheme S2, Supporting Information). Human cervical carcinoma cells (HeLa cells) were used as the model cells. These cells were treated with **3** to facilitate its introduction into the cells, followed by a 1‐day treatment with oleic acid (a potent inducer of lipid droplets) to clearly visualize the tagged cholesterol analog within the cells through its localization on lipid droplets.^[^ ^47^ ^]^ After culturing with oleic acid, the cells were exposed to light for the intracellular photoconversion from **3** to **4**. After photoactivation of the alkyne labeling, the cells were fixed and subsequently stained to visualize the labeling under a microscope. The alkyne moieties on/in the cells were stained by biotinylation with *N*‐(3‐azidopropyl)biotinamide (biotin–N_3_) and subsequent binding to fluorescence‐labeled streptavidin (Cy5–SA) (Figure 4b). In the fluorescence images, granular and fibrous structures were clearly observed throughout the cell, except inside of the nucleus (Figure 4e,f). Consequently, the fluorescence was considered to be labeled uniformly on the lipid membrane covering the intracellular organelles and lipid droplets. This fluorescence image was similar to that of the positive control cells treated with noncaged alkyne‐tagged cholesterol **5** (Figure 4c,d). In the absence of light, fluorescence was scarcely observed in cells treated with caged alkyne‐tagged cholesterol **3** (Figure 4g,h). Quantifying the fluorescent region in each cell through image analysis (Figure 4k) revealed that fluorescence levels were significantly different between cells that were and were not exposed to light. These results strongly suggest that most of the caged alkyne‐tagged cholesterol **3** were converted to**4** through light exposure. Thus, the photocaged alkyne tag was confirmed to be photoconverted to the alkyne moiety, even in living cells, and selectively labeled using CuAAC.

Genetic fusion with fluorescent proteins (FPs) is a widely used approach in intracellular molecular imaging because it enables noninvasive imaging through genetic transduction. Miyawaki et al. developed a photoactivatable FP to study protein dynamics in their pioneering work.^[^ ^1^ ^]^ This is the first study that visualizes real‐time changes in the intracellular localization of phosphorylated proteins; however, the brightness of the FPs is often insufficient to track weakly expressed proteins. FPs fused with 10–15 epitope tags, known as “spaghetti monster” FPs, were developed to track such weakly expressed proteins by additionally immunostaining them with strongly fluorescent chemical probes.^[^ ^47^ ^]^ However, these epitope tags are not photoactivatable. In addition, genetic tagging methods are limited to protein visualization. Thus, conventional gene‐engineering‐based systems have limitations in terms of both detection sensitivity and range of applicable biomolecules.

In contrast, chemically synthesized tags can be applied to a wider range of biomolecules. As demonstrated in this study, they can be directly incorporated into small biomolecules using synthetic approaches. In addition, through semi‐synthetic approaches, such as protein‐ligand binding^[^ ^7^, ^8^ ^]^ and sortagging,^[^ ^11^, ^12^ ^]^ they can be modified on specific proteins in/on living cells. Accordingly, the proposed photoactivatable alkyne tag has the potential to expand the application range of photoinduced molecular imaging from small molecules to specific proteins. Furthermore, in this study, tagged molecules were detected by labeling them with a fluorescent probe via CuAAC‐mediated biotinylation. Unlike gene‐engineering‐based methods, this approach uses various synthetic fluorophores through chemical modification or the ligand binding of streptavidin. Therefore, the range of emission and excitation wavelengths can be greatly expanded, even in the near‐infrared region,^[^ ^48^ ^]^ and fluorescence intensities can be significantly enhanced using quantum dots.^[^ ^49^ ^]^ Further, fluorescent dyes essential for high‐resolution microscopy techniques, such as stochastic optical reconstruction microscopy and photoactivated localization microscopy, can be potentially used.^[^ ^50^ ^]^

## 3 Conclusion

We developed a photoactivatable linear alkyne tag by photo‐caging the hydrazone intermediate in the Eschenmoser–Tanabe reaction. The unstable intermediate was stabilized by modification with a photodegradable protective group. This caged intermediate was photoconverted to an alkyne moiety in an aqueous solution. Accordingly, a photoactivatable alkyne tag was incorporated into a cholesterol analog, and it was introduced into living cells, followed by light irradiation. The photoactivated alkyne tag of the cholesterol analog was microscopically detected in the light‐irradiated cells after CuAAC‐mediated biotinylation and treatment with fluorescence‐labeled streptavidin. This result demonstrates that the proposed alkyne tag successfully achieved the intracellular photoactivation of the alkyne, leading to subsequent bioorthogonal fluorescence labeling for molecular imaging. This photoactivatable alkyne tag offers the potential to expand the application range of photoinduced fluorescence molecular imaging. In addition, it broadens the read‐out modalities by utilizing various probes beyond fluorescent molecules. In principle, activated alkynes can be labeled with metal nanoparticles for electron microscopy^[^ ^51^ ^]^ or with [^18^F] fluoride complexes for positron emission tomography imaging.^[^ ^52^ ^]^ Thus, the proposed photoactivatable alkyne tag is a promising tool for achieving the photoinduced spatiotemporal imaging of various molecules of interest across multiple scales, from intracellular localization to distribution in tissues and the body.

## Conflict of Interest

The authors declare no conflict of interest.

## Supporting information

Supplementary Material

## Data Availability

The data that support the findings of this study are available from the corresponding author upon reasonable request.
