# Supplementary material for: Photoactivatable Alkyne Tag for Photolabeling Biomolecules in Living Cells
Source: Chembiochem. 2025 May 26;26(17):e202500190. doi: 10.1002/cbic.202500190 (PMC12442220; doi:10.1002/cbic.202500190)
Supplement: Supplementary file 1 — Supplementary Material [file CBIC-26-e202500190-s001.pdf]

Supplementary Information

Photoactivatable alkyne tag for photolabeling of biomolecules in living cells

Yuki Umeda[1], Hao Zhu[2], Satoshi Yamaguchi[2],* Sho Nakamura[1], Masato Takada[1], Shin Izuta[1], and Akimitsu Okamoto[1,]*

[1]*Department of Chemistry and Biotechnology, Graduate School of Engineering, The University of Tokyo, 7-3-1 Hongo, Bunkyo-ku, Tokyo 113-8656, Japan*

[2]*SANKEN, Osaka University, 8-1 Mihogaoka, Ibaraki-shi, Osaka 567-0047, Japan*

* Corresponding authors: Satoshi Yamaguchi, SANKEN, Osaka University, 8-1 Mihogaoka, Ibaraki-shi, Osaka 567-0047, Japan. Tel.: +81-6-6879-8465, E-mail: syamaguchi@sanken.osaka-u.ac.jp

Akimitsu Okamoto, Department of Chemistry and Biotechnology, Graduate School of Engineering, The University of Tokyo, 7-3-1 Hongo, Bunkyo-ku, Tokyo 113-8656, Japan. Tel.: +81-3-5841-8701, E-mail: okamoto@chembio.t.u-tokyo.ac.jp

1. Materials and general procedures

1-1. Materials

Unless otherwise specified, the chemicals were of analytical grade and were used without further purification. P-toluenesulfonyl hydrazide (T0286), 4-nitrophenyl chloroformate (C1400), p-toluenesulfonic acid (T0267), biotin-azide (A2523), and sodium ascorbate (A0539) were from Tokyo Chemical Industry Co., Ltd. (Tokyo, Japan). Triethylamine (202-02646), propylamine (169-27962), 4-dimethylaminopyridine (042-19212), toluene (204-01866), DMEM medium (041-29775), oleic acid (151-03403), paraformaldehyde (168-20955), copper(II) sulfate (030-04442) and aminoguanidine hydrochloride (328-26432) were from Fujifilm Wako Pure Chemical Corp. (Osaka, Japan). 4,5-Dimethoxy-2-nitrobenzyl chloroformate (420069-1G), methyl-β-cyclodextrin (C4555-1G), Triton(TM) X-100 (X100-100ML), tris(3-hydroxypropyltriazolylmethyl)amine (THPTA) (762342-100MG), Cy5-streptavidin (GEPA45001) were from Sigma-Aldrich Japan (Tokyo, Japan). Dichloromethane (22414-94), chloroform (08401-94) and Penicillin Streptomycin (09367-34) were from NACALAI TESQUE, INC. (Kyoto, Japan). N-(3-Azidopropyl)biotinamide (biotin-N_3) (HY-129832) was from Cosmo Bio Co. Ltd (Tokyo, Japan). Glass bottom dish (150680) was from Thermo Fisher Scientific K. K. (Tokyo, Japan). Fetal bovine albumin (FBS) was from Nichirei Bioscience (Tokyo, Japan). Dulbecco's phosphate-buffered saline (PBS) was from Shimadzu Diagnostics (Tokyo, Japan).

1-2. General procedures

NMR chemical shifts are reported in ppm downfield of tetramethylsilane using a residual solvent as an internal reference. NMR spectra were recorded using Avance 600 (600 MHz; from Bruker Japan Co., Yokohama, Japan), ECA-600 (600 MHz, JEOL), and ECA-400 (400 MHz, JEOL). High-resolution mass spectra were measured on a LTQ Orbitrap XL (THERMO) equipped with electron spray ionization (ESI). Ultrapure grade water (milli-Q water) was obtained by filtration of deionized water with a Millipore system (from Millipore Japan, Tokyo, Japan). Light irradiation was performed with an ultraviolet (UV) irradiator (LAX-102 or REX250, from Asahi Spectra Co., Ltd., Tokyo, Japan) equipped with a cylindrical lens through a bandpass filter (wavelength: 365 ± 5 nm).

2. Synthesis of photocaged alkyne precursor 1

Photocaged alkyne precursor **1** was newly synthesized in the following procedures (Scheme **S1**). Compounds **6** and **7** were synthesized according to previous literature[S1] with slight modifications.

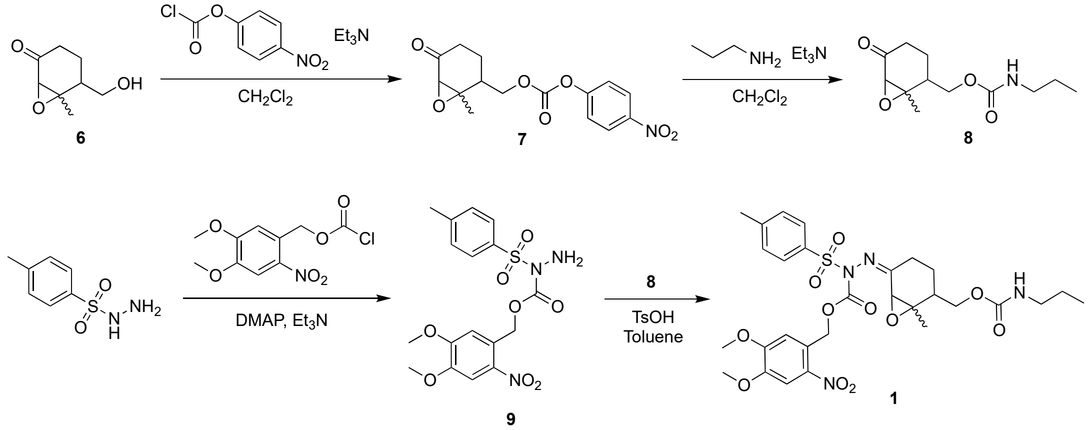

Scheme S1. Synthetic scheme of Photocaged alkyne precursor **1**. The wavy bonds depict stereogenic centers of unknown configuration.

Compound 7. Compound **6** (0.750 g, 4.80 mmol, 1.00 eq) was mixed with 4-nitrophenyl chloroformate (1.70 g, 8.41 mmol, 1.7 eq) in 25 ml of anhydrous CH_2Cl_2. The reaction mixture was stirred on ice and dry triethylamine (1.0 ml, 1.6 eq) was added slowly. The reaction mixture is stirred at 0 °C for 16h. Then, the reaction mixture was evaporated, and a white solid was gained. For purification, the product was solved in 50 ml of ethyl acetate, and washed 6 times with 30 ml of brine. After dried up with sodium sulfate overnight, a yellow viscous liquid was gained with filtration and vacuum distillation. Finally, column chromatography (hexane/ethyl acetate: 4/1) afforded a yellow oil (1.32 mg, yield: 85.5%). ^{1}H-NMR: (600 MHz, $CDCl_3$) δ: 8.29 (d, 2H), 7.39 (d, 2H), 4.38-4.54 (m, 2H), 3.20 (s, 1H), 2.62-2.68 (m, 1H), 2.40-2.52 (m, 1H), 2.26-2.38 (m, 2H), 1.74-1.84 (m, 1H), 1.56 (s, 3H).

Compound 8. Compound **7** (45 mg, 0.20 mmol, 1.00 eq) was mixed with propylamine (82 mg, 0.82 mmol, 4.1 eq) in 10 ml of anhydrous CH_2Cl_2. The reaction mixture was stirred on ice and dry triethylamine (800 μl) was added slowly. The reaction mixture was stirred on ice for 24 h. Then, the reaction mixture was evaporated, and an orange viscous liquid was gained. After, silica column chromatography (hexane/ethyl acetate: 1/1) afforded a yellow viscous liquid (69 mg, yield: 70.7%).

^{1}H-NMR (400 MHz, $CDCl_3$) δ 8.18 (d, J = 9.2 Hz, 1H), 6.94 (d, J = 9.2 Hz, 1H), 4.41 (m, 1H), 4.31-4.19 (m, 1H), 3.19 (m, 2H), 3.02 (s, 1H), 2.58 (m, 1H), 2.30 (m, 1H), 2.11 (m, 1H), 1.94 (m, 1H), 1.76 (m, 1H), 1.59-1.46 (m, 5H), 0.93 (t, J = 7.2 Hz, 3H).

Compound 9. *p*-Toluenesulfonyl hydrazide($TsNHNH_2$) (0.104 g, 0.54 mmol, 1.00 eq), 4,5-Dimethoxy-2-nitrobenzyl chloroformate (NVOCCl) (0.16 g, 0.65 mmol, 1.2 eq), CH_2Cl_2 (16 mL), TEA(0.3 ml, 2.1mmol, 3.90 eq), and 4-dimethylaminopyridine (12.5 mg, 0.13 mmol, 0.24 eq) were mixed and stirred for 1.5h on ice. The mixture was extracted three times with CH_2Cl_2 (15 mL) and $NaHCO_3$ (15 ml). Anhydrous Na_2SO_4 is added to the organic layers. After filtration and evaporation, it was purified by silica column chromatography (hexane/ethyl acetate/cloroform = 2/3/3) to get pale-yellow powder (0.142g, yield: 62%).
^{1}H-NMR (400 MHz, $CDCl_3$) δ 7.85 (d, J = 8.8 Hz, 2H), 7.77 (s, 1H), 7.58 (s, 1H), 7.34 (d, J = 8.4 Hz, 2H), 5.69 (s, 2H), 4.38 (s, 2H), 4.05 (s, 3H), 3.98 (s, 3H), 2.45 (s, 3H).

Photocaged alkyne precursor 1. Compound **8** (22 mg, 0.091 mmol, 1.00 eq) was mixed with p-toluenesulfonic acid (2.0 mg, 0.011 mmol, 0.16 eq), and compound **9** (28mg, 0.065 eq, 1.01 eq) in 10 ml of toluene with Dean-Stark apparatus filled with 20 ml of toluene. The reaction was conducted under 120 °C for 1.5 h. After evaporation, gained crude was purified with silica column chromatography (hexane/ethyl acetate: 1/1) and finally light-yellow viscous material was gained (14 mg, yield: 33%).
^{1}H-NMR (400 MHz, $CDCl_3$) δ 7.90 (d, J = 8.4 Hz, 2H), 7.74 (s, 1H), 7.35 (s, 1H), 7.31 (d, J = 8.4 Hz, 2H), 5.57 (s, 2H), 4.37 (m, 1H), 4.17 (m, 1H), 4.00 (s, 3H), 3.96 (s, 3H), 3.50 (s, 1H), 3.16 (q, 6.4 Hz, 2H), 2.69 (m, 1H), 2.43 (s, 3H), 1.66 (m, 2H), 1.56 (m, 7H), 0.93 (t, J = 7.2 Hz, 3H). ^{13}C-NMR (150 MHz, $CDCl_3$) δ 181.9, 156.2, 154.2, 149.9, 148.3, 145.6, 139.1, 134.1, 129.7, 128.6, 126.6, 109.8, 108.1, 66.3, 64.4, 61.6, 60.2, 56.9, 56.5, 42.8, 37.8, 25.3, 23.2, 21.7, 21.0, 20.9, 11.2. HR-ESI MS *m/z* calcd. For $C_{29}H_{36}N_4O_{11}SNa$ $[M+Na]^+$ 671.1993, found 671.1984.

3. Synthesis of tagged cholesterol analogs

Photocaged alkyne tagged cholesterol analog **3** was newly synthesized in the following procedures (Scheme **S2**).

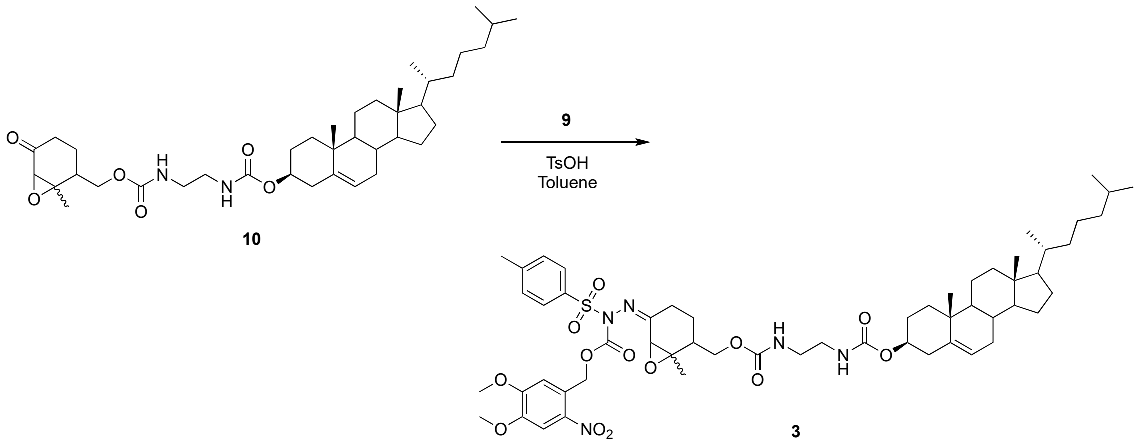

Scheme S2. Synthetic scheme of Photocaged alkyne tagged cholesterol analog **3**. The wavy bonds depict stereogenic centers of unknown configuration.

Compound 10. Compound **10** was synthesized as previously reported.[S1]

Photocaged alkyne tagged cholesterol analog 3. Compound **10** (30 mg, 0.045 mmol, 1.00 eq) was mixed with *p*-toluenesulfonic acid (1.6 mg, 0.0084 mmol, 0.18 eq), and compound **9** (24mg, 0.056 eq, 1.25 eq) in 20 ml of toluene with Dean-Stark apparatus filled with 20 ml of toluene. The reaction was conducted under 120 °C for 4 h. After evaporation, purification was conducted with silica column chromatography (hexane/ethyl acetate: 1/1) and yellow solid was gained (12.3 mg, yield: 25%).
^{1}H-NMR: (600 MHz, $CDCl_3$) δ: 7.90 (d, J = 9.6 Hz, 2H), 7.76 (s, 1H), 7.32 (d, J = 9.6 Hz, 2H), 6.00 (s, 1H), 5.60 (m, 2H), 5.30 (m, 1H), 5.09 (s, 1H), 4.62 (m, 1H), 4.44 (m, 1H), 4.04 (s, 3H), 3.98 (s, 3H), 3.66 (s, 1H), 3.26 (s, 4H), 2.63 (m, 1H), 2.44 (s, 3H), 2.39 (s, 1H), 2.26-0.91 (m, 42H), 0.87 (d, J = 2.4 Hz, 3H), 0.86 (d, J = 3.0 Hz, 3H), 0.67 (s, 3H). HR-ESI MS *m/z* calcd. For $C_{56}H_{79}N_5O_{13}SNa$ $[M+Na]^+$ 1084.5287, found 1084.5294.

4. Evaluation of the alkyne formation with ^{1}H-NMR spectroscopy

An aliquot of the photocaged alkyne precursor **1** (416 nmol) was dissolved in 0.5 mL of chloroform-d_3 and its ^{1}H-NMR spectrum was recorded. After solvent evaporation,

compound **1** was re-dissolved in 208 μL of 1:1 (v/v) mixture of acetonitrile (MeCN) and distilled water in a glass vial to give a final concentration of 2 mM. The solution was irradiated from below the vial using a REX250 light source. Since the vial material attenuates UV light, we measured the light intensity through the bottom of the vial. Upon irradiating the sample with 40 J/cm^2 (22 mW/cm^2 for 1818 s) of 365 nm light, the solvent was evaporated, and the photolysis residue was re-dissolved in 0.5 mL of chloroform-d_3 for subsequent ^{1}H-NMR analysis. As a positive control, compound **2** was directly dissolved in chloroform-d_3.

5. Absorbance measurement after various doses of light

A solution of photocaged alkyne precursor **1** in the mixture of acetonitrile (MeCN) and distilled water (1 : 1) was prepared at the concentration of 0.10 mM in a glass tube with the flat bottom. The solution was exposed to light at various doses of light from 0 to 10 J/cm^2 from below the tube bottom as described above. The absorbance spectra were measured at 25 °C with a UV/vis spectrometer (V-550, from JASCO Co. Ltd, Tokyo, Japan).

6. Intracellular photoactivation of photocaged alkyne tag

Human cervical carcinoma cells (HeLa cells) were purchased from the RIKEN BioResource Research Center (Tsukuba, Japan). HeLa cells were suspended at 5 × 10^4 cells/mL in DMEM supplemented with 10% (v/v) FBS and 0.5% penicillin/streptomycin (culture medium). 1 mL of the cell suspension was added into 35 mm glass-bottom dish. The dishes were incubated overnight at 37°C under 5% CO2. Then, they were washed once with 1 mL of Dulbecco's phosphate-buffered saline (PBS). 100 μL of the solutions of either 1 mM photocaged alkyne-tagged cholesterol **3** or uncaged alkyne-tagged cholesterol **5** (in PBS including 10 mM Methyl-β-cyclodextrin and 5% chloroform) were added into the cell culture dishes, followed by incubation for 10 minutes at 37°C. The dishes were washed once with the culture medium, and then replaced with the culture medium containing 200 μM of oleic acid, followed by incubation overnight at 37°C.

The dishes were washed once with 1 mL of PBS and exposed to light at 10 J/cm^2 from below the dish as described above. Afterward, the dishes were washed once with PBS, and cells were fixed with 1 mL of 3% PFA in PBS (with 5% sucrose) for 15 minutes at room temperature. The dishes were washed once with PBS. Then, 1 mL of 1% TritonX in PBS was added, and the dishes were incubated for 30 minutes at room temperature for

permeabilization. Afterward, the dishes were washed once with PBS. Following this, the reaction mixture for biotinylation was added into the dish, followed by incubation for 20 minutes at 4°C. Here, the reaction mixture for biotinylation is the PBS solution including Biotin-N_3 (final conc. 50 μM), $CuSO_4$ (50 μM), THPTA (250 μM), aminoguanidine hydrochloride (1 mM) and sodium ascorbate (2.5 mM). After incubation, the dishes were washed twice with PBS, and then, a Cy5-Streptavidin solution (1 μM in PBS) was added into the dishes, followed by incubation for 20 minutes at room temperature. After wash with 1 mL of PBS three times, cells were observed using a confocal laser scanning microscope (LSM 510, from Carl Zeiss, Jena, Germany). The fluorescence image was analyzed with ImageJ (NIH, MD, USA). The amount of cholesterol in each cell was evaluated with the region of fluorescence intensity over the threshold, like as previous report detecting fatty acid[S2]. The ratio of fluorescent region was calculated by dividing the fluorescent region with the whole cell size.

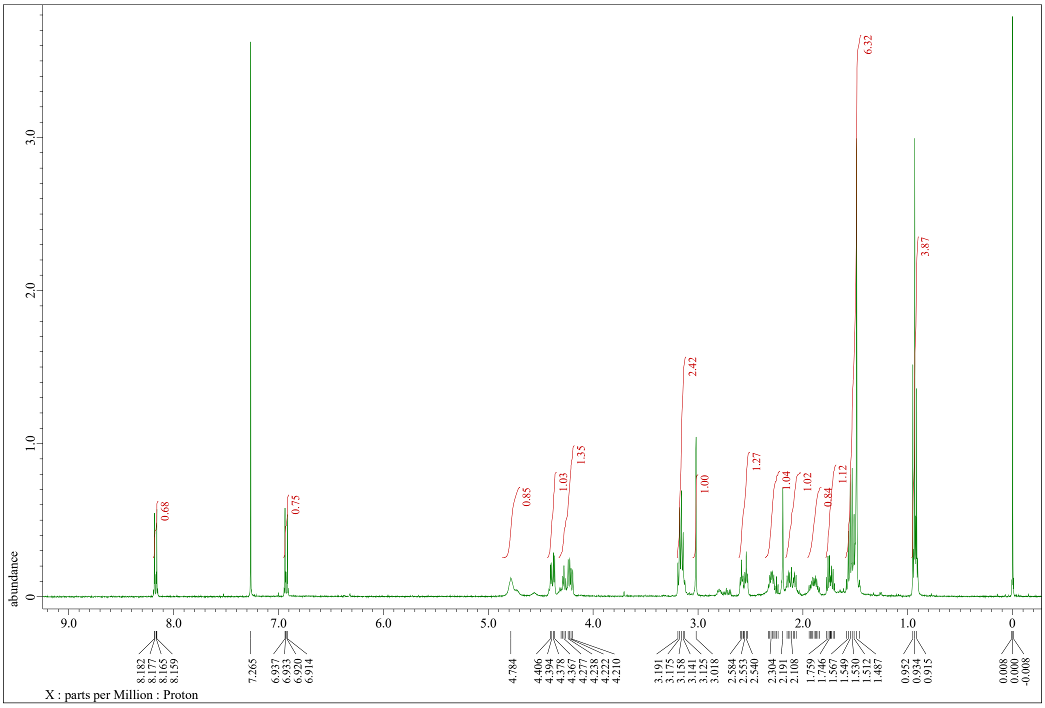

Figure S1 ^{1}H-NMR of compound **8**.

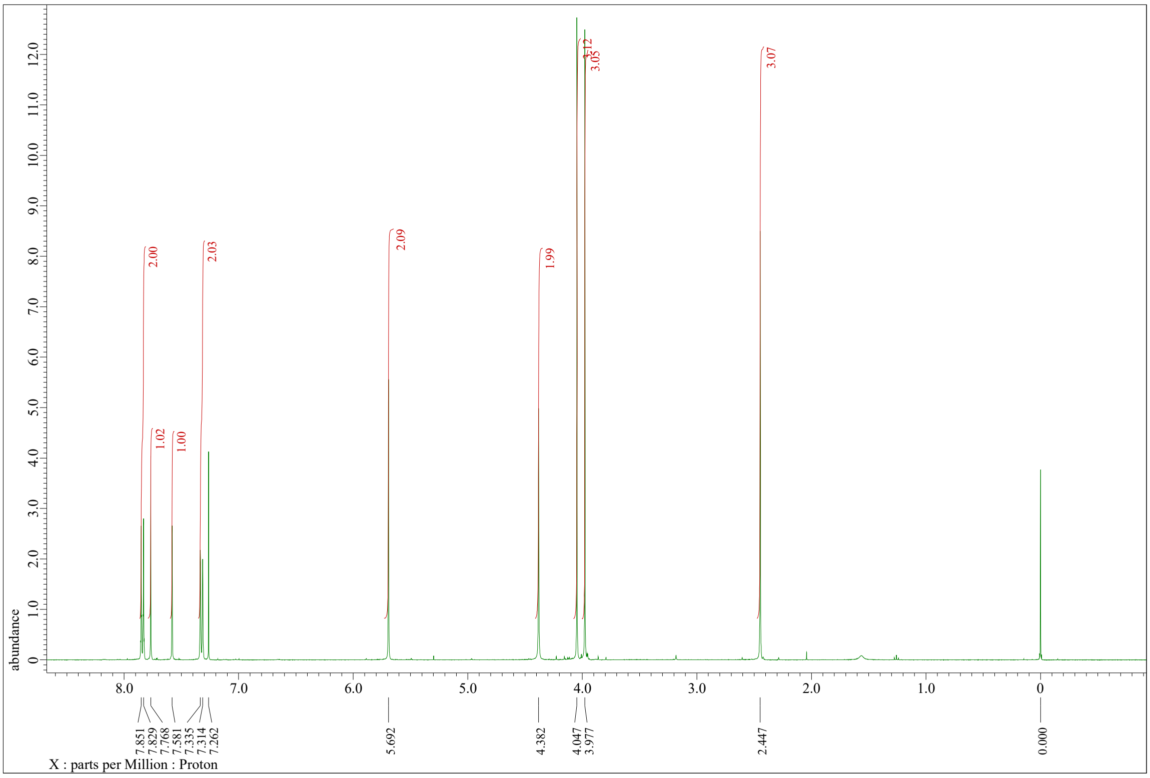

Figure S2 ^{1}H-NMR of compound **9**.

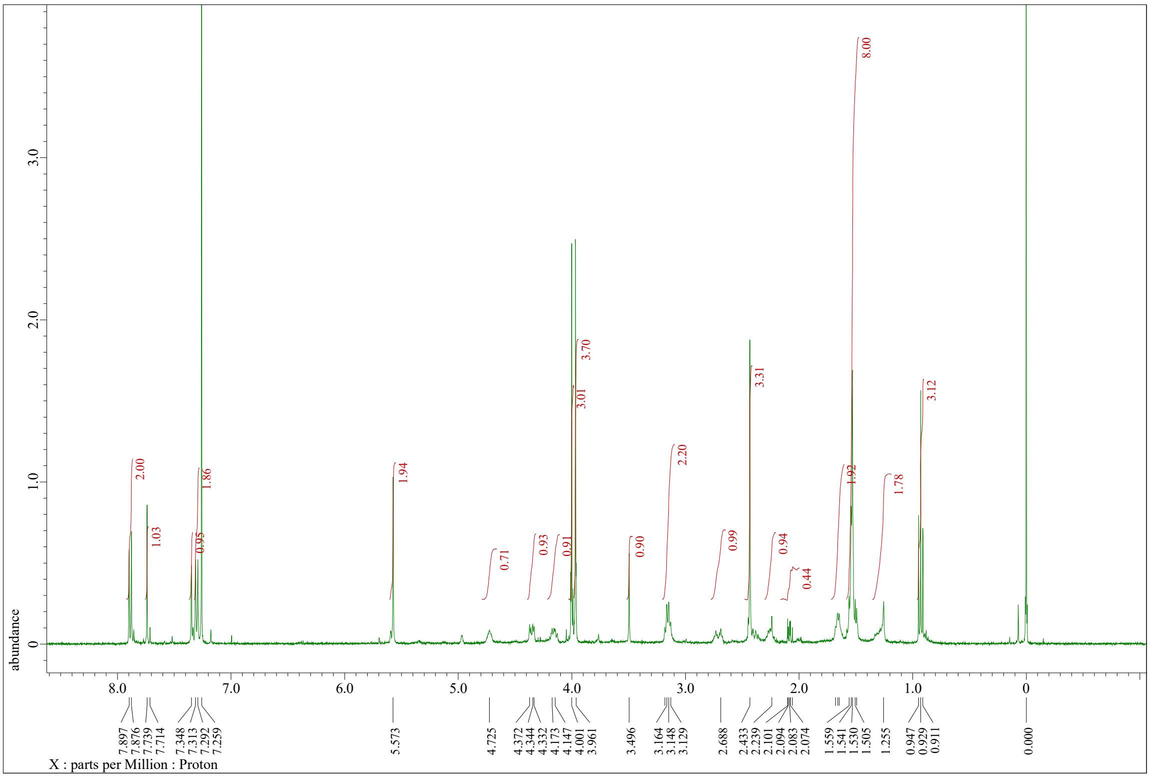

Figure S3 ^{1}H-NMR of compound **1**.

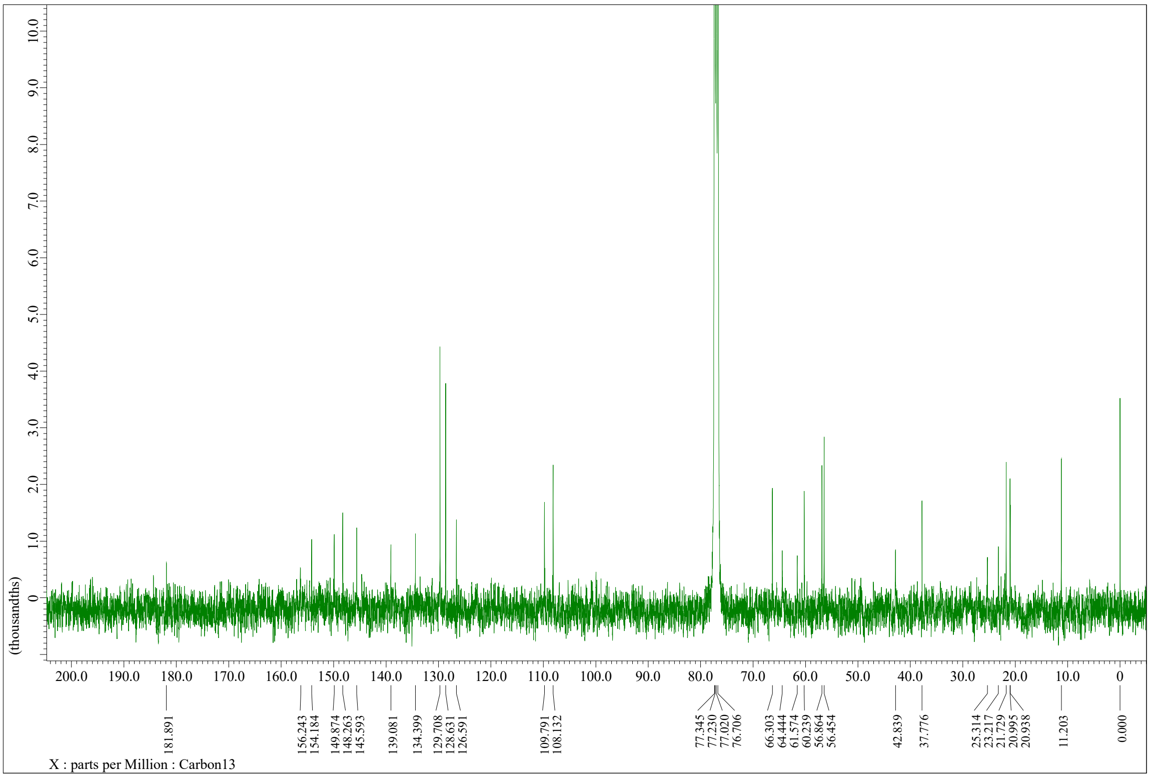

Figure S4 ^{13}C-NMR of compound **1**.

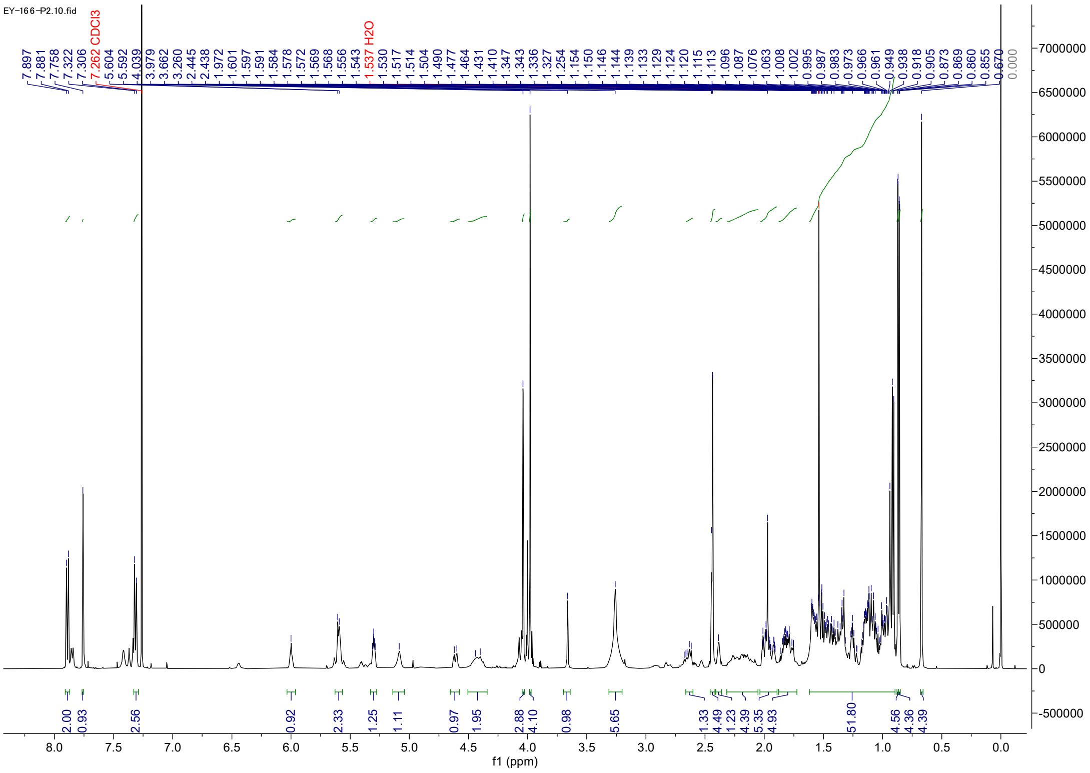

Figure S5 ^{1}H-NMR of compound **3**.

7. References

S1. S. Yamaguchi, M. Ura, S. Izuta, A. Okamoto, *Bioconj. Chem.*, 2016, **27**, 1976-1980.

S2. W. Cui, A. Sathyanarayan, M. Lopresti, M. Aghajan, C. Chen, D. G. Mashek, *Autophagy*, 2021, **17**, 690-705.
